# Tricho- and atrichoblast cell files show distinct PIN2 auxin efflux carrier exploitations and are jointly required for defined auxin-dependent root organ growth

**DOI:** 10.1093/jxb/erv282

**Published:** 2015-06-03

**Authors:** Christian Löfke, David Scheuring, Kai Dünser, Maria Schöller, Christian Luschnig, Jürgen Kleine-Vehn

**Affiliations:** Department of Applied Genetics and Cell Biology, University of Natural Resources and Life Sciences (BOKU), Muthgasse 18, 1190 Vienna, Austria

**Keywords:** Atrichoblast, auxin, epidermal patterning, PIN2, trafficking, trichoblast.

## Abstract

The phytohormone auxin is a vital growth regulator in plants. In the root epidermis auxin steers root organ growth. However, the mechanisms that allow adjacent tissues to integrate growth are largely unknown. Here, the focus is on neighbouring epidermal root tissues to assess the integration of auxin-related growth responses. The pharmacologic, genetic, and live-cell imaging approaches reveal that PIN2 auxin efflux carriers are differentially controlled in tricho- and atrichoblast cells. PIN2 proteins show lower abundance at the plasma membrane of trichoblast cells, despite showing higher rates of intracellular trafficking in these cells. The data suggest that PIN2 proteins display distinct cell-type-dependent trafficking rates to the lytic vacuole for degradation. Based on this insight, it is hypothesized that auxin-dependent processes are distinct in tricho- and atrichoblast cells. Moreover, genetic interference with epidermal patterning supports this assumption and suggests that tricho- and atrichoblasts have distinct importance for auxin-sensitive root growth and gravitropic responses.

## Highlight

PIN2 shows distinct trafficking and vacuolar turnover in neighbouring tricho- and atrichoblast cell files. Differential abundance of PIN2 in the root epidermis could have developmental importance for root gravitropism.

## Introduction

Plant cells do not alter the relative position to each other. Therefore, symplastic growth regulation is typical for plant tissue expansion (Priestley, 1930; Erickson, 1986). This implies that tissue growth occurs above the level of single cells (supra-cellular growth regulation). Despite its importance in plants, very little is known about how neighbouring tissue growth is integrated into organ growth. The epidermis represents the outermost tissue and has outstanding importance for organ growth (Savaldi-Goldstein *et al.*, 2007), suggesting that epidermal growth regulation is able to impact on deeper tissues.

The phytohormone auxin has been suggested to regulate root organ growth in epidermal cell layers (Luschnig *et al.*, 1998). Intercellular auxin transport is particularly central to asymmetric growth regulation, ensuring gravitropic root growth. The directional transport of auxin is ensured by plasma membrane-localized PIN-FORMED (PIN) auxin carrier proteins (Wisniewska *et al.*, 2006). Gravity perception in the columella cells at the very tip of the root regulates the partially polarized deposition of PIN3, allowing re-location of auxin transport in the direction of the gravity vector (Friml *et al.*, 2003; Kleine-Vehn *et al.*, 2010). The possibly asymmetric flux of auxin is picked up shoot-wards by PIN2 in the epidermis, establishing asymmetric auxin flows into the elongation zone (Luschnig *et al.*, 1998; Kleine-Vehn *et al.*, 2008). Differences in auxin levels at the upper and lower flanks of the root will ultimately lead to differential growth control, determining root bending towards the gravity vector.

Polar vesicle trafficking, low lateral mobility in the plasma membrane, and localized internalization appears to ensure PIN2 localization at the shoot-ward (apical) epidermal cell side (Kleine-Vehn *et al.*, 2011). PIN proteins constitutively cycle between the plasma membrane and early endosomal compartments (Geldner *et al.*, 2001). The fungal toxin and specific ARF-GEF vesicle trafficking inhibitor Brefeldin A (BFA) allows the dissection of PIN protein recycling back to the plasma membrane (Geldner *et al.*, 2001, 2004) and also interferes with PIN protein transition into the lytic vacuole for degradation (Kleine-Vehn *et al.*, 2008).

Besides the importance of the epidermal cell file for auxin-dependent root growth, little is known about how growth is coordinated within this tissue. The root epidermis is regularly spaced between tricho- and atrichoblasts in *Arabidopsis thaliana*. Trichoblasts develop root hairs in the differentiation zone, while atrichoblasts become non-hair cells (Dolan *et al.*, 1993; Dolan, 2001; Scheres *et al.*, 2002). In the root meristem, trichoblast cells are shorter than atrichoblast cells and its cellular size regulation is interdependent (Löfke *et al.*, 2013). However, the contribution of these neighbouring cells to auxin-dependent root organ growth remains to be addressed.

## Material and methods

### Plant material and growth conditions

*Arabidopsis thaliana* ecotype *Columbia (Col-0*) was used. The plant lines have been described previously: PIN2::GUS (Benkovà *et al.*, 2003); PIN2::PIN2-GFP(1) (Abas *et al.*, 2006); PIN2::PIN2-GFP(2) (Xu and Scheres, 2005); PIN2-VENUS, *pin2* ^12K-R^-VENUS, and PIN2-ubq-VENUS (Leitner *et al.*, 2012); ABCB19::ABCB19-GFP (PGP19) (Mravec *et al.*, 2008); *wer myb23* (Lee and Schiefelbein, 1999, 2001); and *cpc try* (Schellmann *et al.*, 2002). Seeds were stratified at 4 °C for 2 d in the dark and grown on vertically orientated half-strength Murashige and Skoog (MS) (1% sucrose) medium plates under a long-day regime (16/8h light/dark) at 20–22 °C.

### Chemicals

All chemicals were dissolved in DMSO and were applied in solid or liquid half-strength MS-medium. 1-Naphthaleneacetic acid (NAA) was obtained from Duchefa, Brefeldin A (BFA) and wortmannin (WM) from Cayman Chemical, propidium iodide (PI) from Sigma, and FM4-64 from Invitrogen (Molecular probes).

### Phenotype analysis

Gravitropism assays: seven-day-old seedlings were grown vertically and gravi-stimulated by turning the plates by 135° for an additional 12h stimulation in the dark. After turning by 135°, roots that showed a normal gravitropic response grew in the diagonal of the Petri dish and deviations of the response could be monitored. All gravitropically stimulated roots were assigned to one of four 15° sectors on a gravitropism diagram. The length of the bars in the diagram represents the percentage of seedlings assigned to the respective sector (*n*=12). For time -course experiments, 7-d-old seedlings were gravi-stimulated by a 90° rotation and monitored in a lightproof box equipped with a spectrum-enhanced camera (EOS035 Canon Rebel T3i) modified by Hutech technologies by a built-in clear wideband-multicoated filter and operated by EOS utility software. Embedded LED infrared diodes (880nm) dispense light for illuminating the samples. The angle of the root tips were recorded after 2, 4, 8, 12, and 24h and were analysed with the ImageJ software (http://rsb.info.nih.gov/ij/). Eight to twelve roots per line were measured in three individual experiments.

Root growth assay: seedlings were germinated on vertically oriented half-strength MS plates supplemented with 125nM NAA or DMSO as a solvent control. After 7 d, root length was determined by scanning the seedlings on a flatbed scanner to acquire images suitable for quantification using ImageJ (http://rsb.info.nih.gov/ij/). Fifteen seedlings were measured in three independent experiments.

### Drug treatments

Brefeldin A (BFA) and wortmannin (WM) were both applied in liquid MS-medium and control treatments were performed by using DMSO as the solvent control. Seedlings were treated with 50 μM BFA for 60–90min or with 30 μM WM for 4–5h (see Fig. 2) or for 2h (see Supplementary Fig. S3 at *JXB* online), respectively. Samples were subsequently imaged and quantified by measuring the signal intensity of the BFA or WM compartments, respectively. Five cells each were analysed in five individual roots. The quantifying effects of BFA on root length: *wer myb23*, *cpc try* and, as a control, WER::GFP seedlings were germinated on vertically oriented half-strength MS plates supplemented with 5 μM BFA or DMSO as a solvent control. After 6–7 d, root length was determined by scanning the seedlings on a flatbed scanner to acquire images suitable for quantification using ImageJ (http://rsb.info.nih.gov/ij/). To determine agravitropic growth, the vertical growth index (VGI) was quantified accordingly to Grabov *et al.* (2005). In brief, the shortest distance between the shoot–root junction and the root tip was measured (Ly) and divided by the root length (L). Ten individual seedlings were analysed in four independent experiments.

### Immunolocalization of PIN2

Whole mount immunolocalization was prepared as previously described by Sauer *et al.* (2006). Antibodies were diluted as follows: 1:500 for anti PIN2 and incubated overnight (Abas *et al.*, 2006); 1:500 for CY5-conjugated goat/anti-rabbit secondary antibody (Dianova) and incubated for 3h. Images were quantified by measuring the PM signal in five cells per root in five individual seedlings.

### GUS-stainings

Six-day-old pPIN2::GUS seedlings were fixed for 1h in 95% cold acetone on ice at –20 °C, and subsequently washed in sodium phosphate buffer 0.1M for 1h. The seedlings were then subjected to GUS staining solution (sodium phosphate buffer 0.1M; EDTA 10mM; K_3_FeCN_6_ 0.5mM; K_4_FeCN_6_ 0.5mM; Triton X 100 0.1%; X Gluc 0.1mM) in the dark at 37 °C for 30min. The seedlings were then cleared in 1:3 v/v acetic acid/ethanol for 2h and subjected to a graded ethanol series (70% EtOH, 50% EtOH, and 20% EtOH) for 10min per concentration. After chloral hydrate treatment, the seedlings were mounted on microscope slides. Image acquisition was performed with a light microscope (Leica DM 5500) equipped with a DFC 300 FX camera (Leica).

### Microscopy

For live cell imaging, wherever needed, roots were mounted in a propidium iodide (PI) solution (0.02mg ml^–1^) for counterstaining the cell walls. Endocytosis assay: seven-day-old seedlings were stained in a 2 μM FM4-64 solution (MS-medium) on ice for 5min, followed by a 20min incubation step at room temperature. Samples were immediately imaged and quantified by measuring the plasma membrane signal (to normalize FM4-64 loading) and intracellular signal of the cell. Each experiment consists of five cells per root in five individual seedlings.

Vacuolar accumulation assay: seven-day-old PIN2-GFP seedlings were incubated in the dark for 5h and subsequently imaged to record the PIN2-GFP loaded vacuole (see also: Kleine-Vehn *et al.*, 2008; Scheuring *et al.*, 2015). Due to (possibly) lower turnover rates of ABCB19-GFP compared to PIN2-GFP, time extended dark treatments of 6–7h were used. Images were quantified by measuring the signal in the vacuolar lumen or intracellular signals in five cells per root in five individual seedlings. The quantification method is further defined in the figure legends.

FM4-64 staining of the tonoplast membrane was performed as previously described by Scheuring *et al.* (2015). In brief: seedlings were incubated for 20min in an 8-well-plate containing liquid MS-medium supplemented with 4 μM of FM4-64 and subsequently incubated in darkness for 4–5h in fresh liquid MS-medium. This allows the accumulation of GFP in the vacuolar lumen and FM4-64 incorporation in the tonoplast membrane.

For image acquisition a Leica DM6000 CS, TCS AOBS confocal laser scanning microscope (SP5) was used, equipped with a HCX PL APO CS 63.0×1.20 WATER objective. Fluorescence signals were processed with the Leica software LAS AF 3.1 or with ImageJ (http://rsb.info.nih.gov/ij/) and data were statistically evaluated by Student’s *t* test using graphpad (http://www.graphpad.com/quickcalcs/). PIN2 images were quantified by measuring the signals in five cells per root in five individual seedlings. BFA compartments were quantified by either measuring the intracellular signals or by quantifying the mean grey value of the brightest BFA compartment per cell. Vacuolar PIN2-GFP signals were quantified either by measuring the entire intracellular signals or by quantifying the mean grey value of the brightest vacuolar structure. The respective quantification method is specified in each graph and figure legend. Representative images are shown.

## Results

### Tricho- and atrichoblast cells show distinct PIN2 protein levels at the plasma membrane

**Fig. 1.**
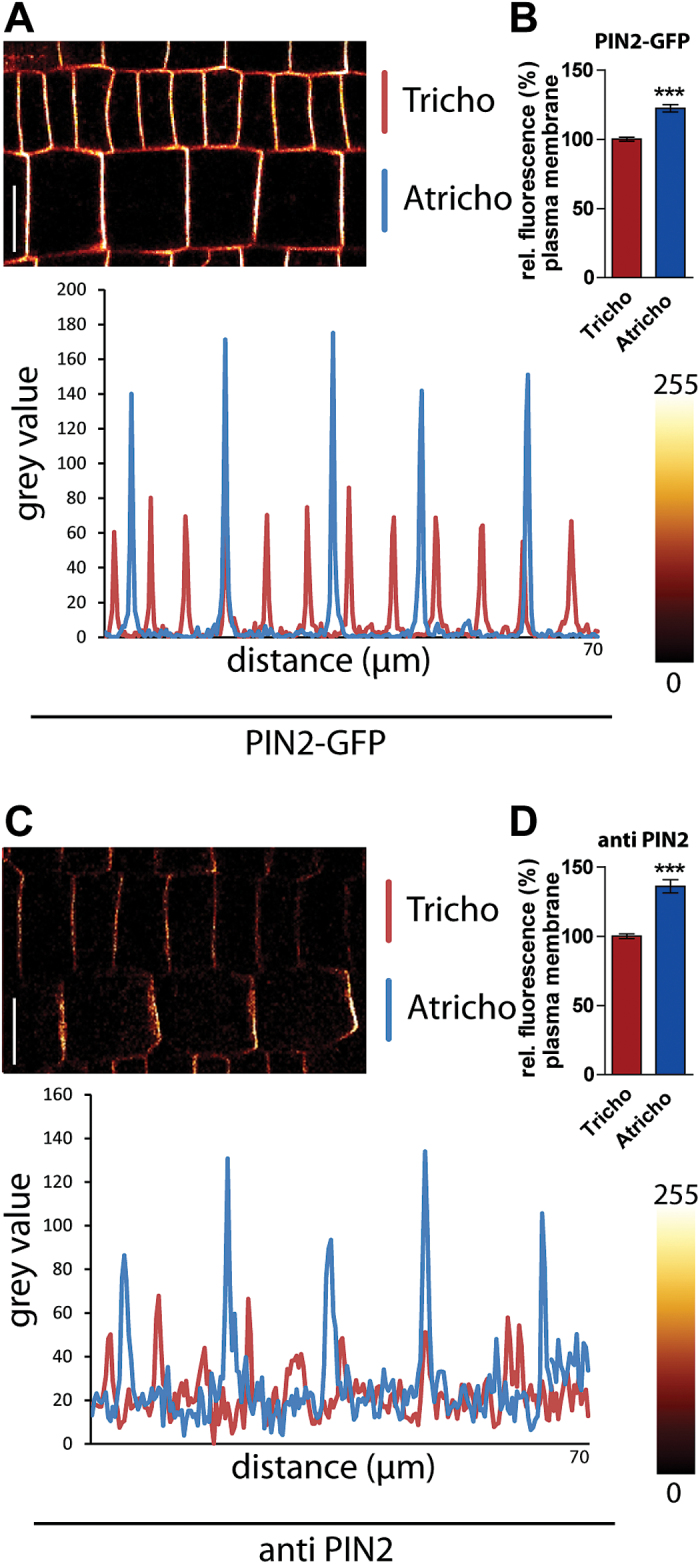
PIN2 protein levels are distinct in neighbouring epidermal cell files. (A) PIN2-GFP-expressing trichoblast and atrichoblast cell files display different levels of PIN2. (B) Atrichoblast cell files have a 20% stronger fluorescence signal at the plasma membrane than trichoblast cell files. A profile of fluorescence intensity (grey value) through the atrichoblast and trichoblast cell files is shown underneath. (C) Immunolocalization studies with a PIN2 antibody confirmed a stronger signal in atrichoblast cell files. A profile of fluorescence intensity through the cell files is shown underneath. (D) Quantification of fluorescence intensity at the plasma membrane revealed a 30% stronger signal at atrichoblast cell files. The data were statistically evaluated using Student’s *t* test. ***P <0.001; *n*=25 cells in five individual roots. Scale bar: 10 μm.

PIN2 auxin efflux carriers are the major root epidermal auxin transport components, ensuring shoot-ward auxin flux and are crucial for gravitropic root growth (Luschnig *et al.*, 1998). To investigate PIN2-dependent auxin transport, functional PIN2-GFP fusion proteins were initially investigated under the endogenous promoter in *Arabidopsis thaliana* (Xu and Scheres, 2005; Abas *et al.*, 2006). The fluorescence intensity of PIN2-GFP at the apical plasma membrane was higher in atrichoblast cells compared to trichoblast cells (Fig. 1A). The fluorescence intensity was approximately 20% higher in these cells (Fig. 1B), suggesting a possible biological importance. To address whether this effect is related to the transgene used, endogenous PIN2 proteins were also investigated. Immunocytochemistry and confocal microscopy of PIN2 on whole mount *Arabidopsis* seedlings also confirmed that endogenous PIN2 has, approximately, a 30% higher protein occurrence in atrichoblast cells compared with trichoblast cells (Fig. 1C, D). In order to address the specificity of our finding on differential PIN2 abundance in tricho- and atrichoblast cells, the non-polar auxin ATP-binding cassette (ABC) transporter, ABCB19-GFP (Mravec *et al.*, 2008), was used as a control. In contrast to PIN2, ABCB19-GFP did not show differential abundance at epidermal plasma membranes (see Supplementary Fig. S1A, B at *JXB* online), suggesting a certain specificity for PIN2 abundance control in these neighbouring cells.

In summary, it is shown that endogenous and transgenic PIN2 proteins show distinct levels in neighbouring epidermal tricho- and atrichoblast cells.

### Vesicle trafficking inhibitor Brefeldin A has differential effects on PIN2 in epidermal cell files

**Fig. 2.**
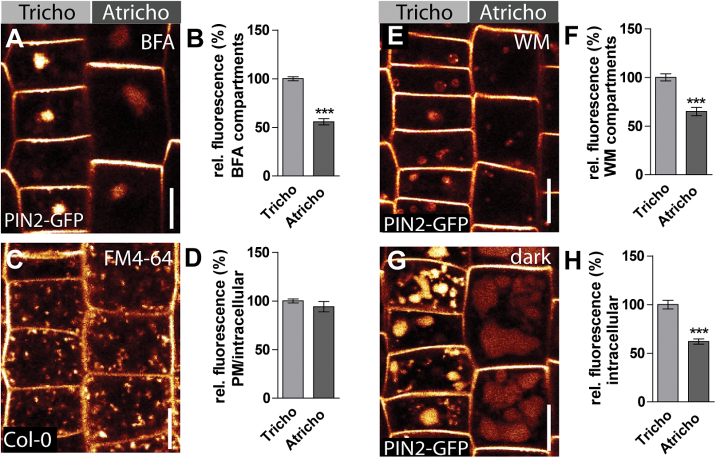
PIN2 displays distinct trafficking in tricho- and atrichoblast cells. (A) BFA treatment (50 μM) of PIN2-GFP plants for 60–90min. (B) Quantification of BFA body signal intensity. Trichoblast cells show brighter BFA bodies than atrichoblast cells. (C) FM4-64 uptake in both cell types. (D) Quantification of plasma membrane (PM) and intracellular FM4-64 signals shows that the general endocytic uptake rate in both cell types is equal. (E) 30 μM wortmannin (WM) treatment for 4–5h of PIN2-GFP expressing plants. (F) Quantification of intracellular signals of WM compartments revealed brighter structures in trichoblast cell files. (G) PIN2-GFP vacuole accumulation assay: PIN2-GFP plants were kept in the dark for 5h and the fluorescence signal in the vacuoles of atrichoblast and trichoblast cells was subsequently imaged. (H) Quantification of intracellular PIN2-GFP signals. Trichoblast cells show a brighter vacuolar signal than atrichoblast cells. The data were statistically evaluated using Student’s *t* test. ***P <0.001; *n*=25 cells in 5 individual roots. Scale bar: 10 μm.

A transcriptional *PIN2* reporter (PIN2::GUS) did not show distinct *PIN2* expression in tricho- and atrichoblast cell files, suggesting that PIN2 trafficking might be distinct in these neighbouring cells (see Supplementary Fig. S2A, B, C at *JXB* online). The vesicle trafficking inhibitor Brefeldin A (BFA) is widely used to affect intracellular vesicle trafficking. BFA application leads to PIN2 accumulation in aggregating endosomes, also called the BFA compartments (Geldner *et al.*, 2001). BFA (50 μM) was applied for 60–90min on PIN2-GFP expressing seedlings (Fig. 2A). Surprisingly, trichoblast cell files, despite showing lower levels of PIN2-GFP at the plasma membrane, displayed stronger PIN2-GFP accumulation into BFA compartments compared with atrichoblast cells (Fig. 2B). However, it was noted that the shape of the BFA compartments often appeared different in tricho- and atrichoblasts. To confirm that the observed differences in fluorescence intensity of PIN2-GFP are not due to optical sectioning of the BFA compartment, defined *z*-stack imaging was performed and maximum projections were subsequently established, which enabled the overall intracellular fluorescence of a given cell to be quantified (see Supplementary Fig. S3A at *JXB* online). Again, a stronger intracellular fluorescence signal of PIN2-GFP was detected in entire trichoblast cells (see Supplementary Fig. S3B at *JXB* online). This set of data suggests that PIN2 proteins show higher intracellular trafficking and/or internalization rates in trichoblasts.

To investigate whether endocytosis is generally enhanced in trichoblast cells, the endocytic tracer FM4-64 was used (Fig. 2C). Exogenous application of FM4-64 leads to the integration of the dye into the outer leaflet of the plasma membrane. Subsequent dye internalization depends on functional endocytosis in *Arabidopsis* (Bolte *et al.*, 2004; Jelinkova *et al.*, 2010). No detectable differences were observed in FM4-64 internalization in tricho- or atrichoblast cell files (Fig. 2D). Therefore, it is assumed that bulk flow endocytosis is not distinct in neighbouring root epidermal cell files.

These data suggest that BFA-sensitive intracellular PIN2 targeting is partially distinct in tricho- and atrichoblast cells.

### PIN2 shows higher lytic turnover in trichoblast cells

Lower PIN2 abundance at the plasma membrane correlated with higher accumulation into BFA compartments in trichoblast cells. Therefore, it was assumed that certain PIN2 trafficking events might lead to reduced PIN2 occurrence at the plasma membrane. BFA inhibits PIN2 recycling to the plasma membrane, but also its transition into the lytic vacuole for degradation (Kleine-Vehn *et al.*, 2008). To address late endocytic vesicle trafficking, wortmannin (WM), a widely used phosphatidylinositol kinase inhibitor (Balla, 1998; Vanhaesebroeck *et al.*, 2001), was used. WM application leads to the swelling of the pre-vacuolar compartments (Wang *et al.*, 2009) and PIN2-GFP proteins readily accumulated into these WM-sensitive structures in both tricho- and atrichoblast cells (Fig. 2E). However, PIN2-GFP accumulation in WM bodies was enhanced in trichoblast cells (Fig. 2F). To address the overall cellular accumulation of PIN2-GFP, defined *z*-stack imaging was performed and the intracellular PIN2-GFP signal in maximum projections was quantified. In agreement with our single section data, PIN2-GFP showed stronger accumulation into WM-sensitive structures in entire trichoblast cells (see Supplementary Fig. S3C, D at *JXB* online). These data suggests that PIN2 constitutively shows higher transit into pre-vacuolar compartments in trichoblasts.

It was then addressed whether lytic degradation of PIN2 might be enhanced in trichoblast cell files. Dark treatments affect the vacuolar degradation of GFP and, hence, can be used to assess the lytic degradation of GFP-tagged plasma membrane proteins (Kleine-Vehn *et al.*, 2008). Dark treatment for 5h of PIN2-GFP transgenic lines was sufficient to inspect strong GFP fluorescence in vacuoles (Fig. 2G). Notably, the fluorescence intensity in these structures was higher in trichoblast cells compared with atrichoblast cells (Fig. 2H). To address whether membrane crowding or sectioning artefacts could impose higher PIN2-GFP fluorescence in trichoblasts, the tonoplast membrane was co-labelled using FM4-64 (Scheuring *et al.*, 2015; see Supplementary Fig. S4A, B, C at *JXB* online). This approach allowed the vacuolar lumen and membrane intensity to be quantified. This co-labelling experiment again showed higher fluorescence intensity within the vacuoles of trichoblast cells (see Supplementary Fig. S4D at *JXB* online), whereas the FM4-64 signal was equal in both cell types (see Supplementary Fig. S3E at *JXB* online).

Based on this finding, it is concluded that membrane crowding does not induce higher fluorescence in trichoblast cells. However, the size of the vacuolar lumen could also impact on our analysis and, hence, the vacuolar turnover of ABCB19-GFP, which showed even protein abundance in tricho- and atrichoblasts was also analysed (see Supplementary Fig. S1A, B at *JXB* online). It has been suggested that ABCB19 shows a relatively stable localization at the plasma membrane (Titapiwatanakun *et al.*, 2009). Therefore, the dark treatments were extended to ensure that the vacuolar turnover of ABCB19-GFP was captured. Following this approach, ABCB19-GFP was clearly detected in lytic vacuoles, but we did not observe any differential intracellular fluorescence intensity of the ABCB19-GFP signal in the vacuoles of tricho- and atrichoblasts (see Supplementary Fig. S1C, D at *JXB* online). These data imply that the stronger accumulation of PIN2-GFP in trichoblasts is due to distinct trafficking events in tricho- and atrichoblasts. Interestingly, the ABCB19 related auxin carrier ABCB4 shows auxin-induced differential turnover in epidermal cells (Kubes *et al.*, 2012), suggesting that not only PIN2 but also other proteins may show differential trafficking in tricho- and atrichoblasts in response to certain stimuli.

This set of data suggests that PIN2-GFP in trichoblast cells is subjected to a higher targeting to the lytic vacuole for degradation. Our data on WM sensitivity and vacuolar accumulation of PIN2-GFP is in agreement with a higher protein turnover of PIN2 in trichoblast cell files.

### Interference with ubiquitin-dependent PIN2 turnover rates disrupts differential PIN2 abundance in tricho- and atrichoblasts

**Fig. 3.**
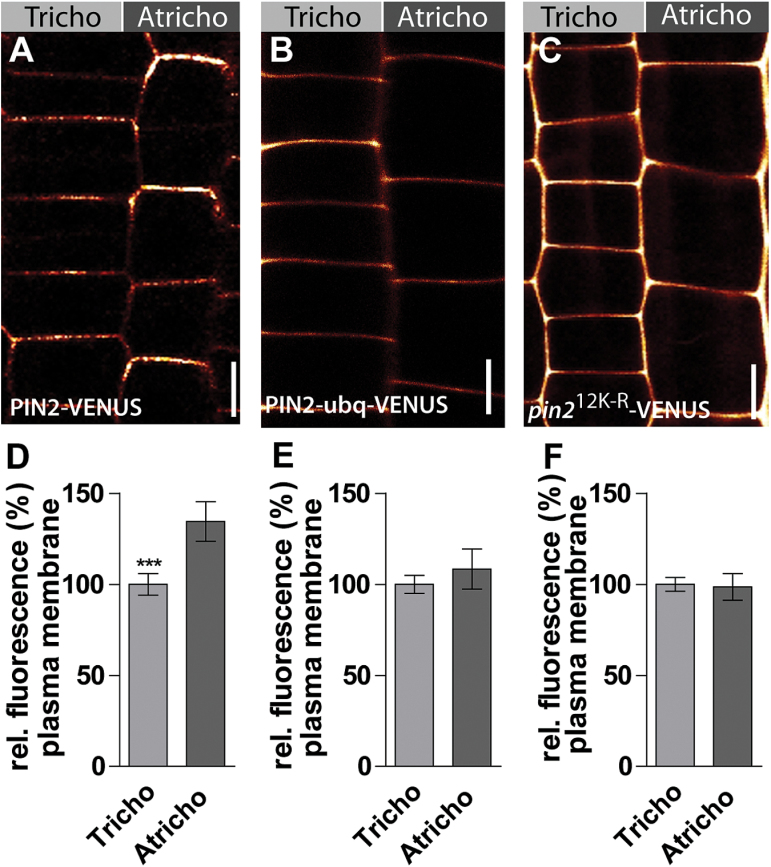
Interference with PIN2 trafficking abolishes differential PIN2 abundance in neighbouring epidermal cell files. (A) PIN2-VENUS as control at the plasma membrane. (B) Ubiquitinated form (PIN2-ubq-VENUS) of PIN2. (C) PM-stabilized form (*pin2* ^12K-R^-VENUS) of PIN2. (D) PIN2-VENUS shows a 30% stronger signal at the plasma membrane of atrichoblast cells. (E) PIN2-ubq-VENUS does not display differences at the plasma membrane in atrichoblast and trichoblast cells. (F) *pin2* ^12K-R^-VENUS also does not display differences in fluorescence intensity at the plasma membrane of atrichoblast and trichoblast cells. The data were statistically evaluated using Student’s *t*-test. ***P <0.001; *n*=25 cells in five individual roots. Scale bar: 10 μm.

Our pharmacological and live-cell imaging approaches suggest that distinct PIN2 turnover rates in tricho- and atrichoblast cell files lead to differential PIN2 protein levels at the plasma membrane in these neighbouring cell files. To assess this hypothesis it was decided to interfere genetically with PIN2 turnover rates. It has been shown that the fusion of PIN2 to ubiquitin (PIN2-ubq-VENUS) leads to its constitutive transport into the vacuole for degradation (Leitner *et al.*, 2012). By contrast, mutations of the potential lysine residues for ubiquitination in the hydrophilic loop of PIN2 (*pin2* ^12K-R^-VENUS) leads to a stabilization at the plasma membrane (Leitner *et al.*, 2012). In the first approach, PIN2 trafficking to the vacuole was expected to be constitutively on, in the latter case, strongly reduced. Using these tools, it was subsequently investigated whether altered PIN2 turnover rates affect differential PIN2 abundance in neighbouring epidermal cells. PIN2-VENUS-expressing control seedlings showed the expected differential PIN2 abundance, showing about 30% higher signals in atrichoblasts (Fig. 3A, D). By contrast, ubiquitin-based destabilization and stabilization of PIN2-VENUS in both cell types abolished the differential protein occurrence (Fig. 3B,E; C,F).

These data support our assumptions that PIN2 proteins show differential degradation rates in tricho- and atrichoblasts, leading to distinct PIN2 abundance at the plasma membrane in these neighbouring tissues.

### Epidermal patterning mutants lack differential PIN2 distribution

PIN2 proteins show distinct trafficking in tricho- and atrichoblast cells, probably leading to distinct turnover rates. To assess whether tricho- and atrichoblast identity triggers differential PIN2 abundance at the plasma membrane, endogenous PIN2 was inspected in tricho- and atrichoblast patterning mutants. Epidermal patterning mutants that already display cell identity defects in the root meristem were used (Löfke *et al.*, 2013).

**Fig. 4.**
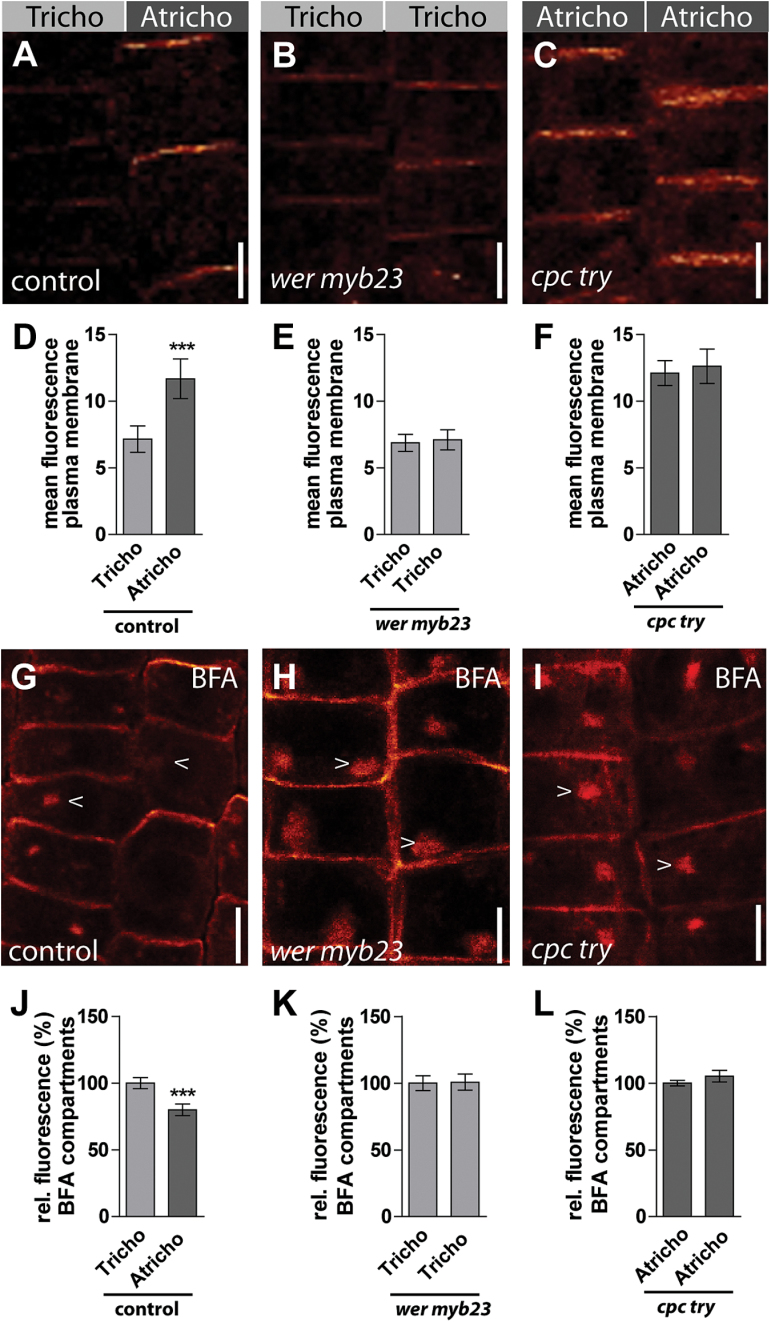
Immunolocalization of PIN2 in epidermal patterning mutants. (A–C) PIN2 signal at the plasma membrane in atrichoblast and trichoblast cells of control plants (A), *wer myb23* (B), and *cpc try* (C) plants. (D) Quantification of the plasma membrane signal of (A) shows a 30% stronger signal in atrichoblast cells compared with trichoblast cells. The mean fluorescence of PIN2 in cell files of *wer myb23* (E) and *cpc try* (F) plants. Settings for confocal image acquisition were kept unchanged for a comparability of absolute signal intensities in (A), (B) and (C). (G) Immunolocalization of BFA-treated control plants. BFA treatment (1 h, 50 μM) leads to a redistribution of PIN2 into BFA bodies. (H, I). In neighbouring *wer myb23* (H) and *cpc try* (I) mutant epidermal cells, PIN2 containing BFA bodies are equal in intensity. (J) Quantification of the fluorescence intensity of BFA bodies in atrichoblast and trichoblast cells shows a stronger signal in the latter. (K, L) Fluorescence intensities of BFA bodies in both cell types are equal in *wer myb23* (K) and *cpc try* (L) plants. The data were statistically evaluated using Student’s *t* test. ***P <0.001; *n*=25 cells in five individual roots. Scale bar: 10 μm.

Werewolf (WER) and MYB23 are partially redundant R2R3 transcription factors required for non-hair-cell fate already in the meristem (Lee and Schiefelbein, 1999; Kang *et al.*, 2009). *wer myb23* double mutants only show trichoblast identity and visibly lost competence for the differential control of PIN2 in neighbouring epidermal cell files (compare Fig. 4A–B; D–E). PIN2 abundance was not only non-differential but, in addition, the mean fluorescence in both neighbouring cell files was notably low and, hence, comparable with trichoblast cells of control plants (Fig. 4E).

CAPRICE (CPC) and TRIPTYCHON (TRY) encode for MYB-related transcription factors with high sequence similarities and act redundantly in position-dependent cell fate determination in the root epidermis (Schellmann *et al.*, 2002). In agreement, *cpc try* double mutant roots have only atrichoblast identity in meristematic, neighbouring epidermal cells (Lee and Schiefelbein, 1999) and showed no visible differential PIN2 abundance at the plasma membrane in neighbouring epidermal cell files (compare Fig. 4A–F). Similar to *wer myb23,* no quantitative difference in PIN2 abundance at the PM could be observed in only atrichoblast bearing *cpc try* double mutants, but the mean fluorescence in all epidermal cells was comparably high, being reminiscent of the atrichoblast cells of control plants (Fig. 4 C, F).

These data suggest that epidermal patterning genes contribute to cell fate-dependent abundance of PIN2 at the plasma membrane.

To test whether the epidermal patterning mutants also lost the differential regulation of intracellular PIN2 trafficking, BFA treatments were performed on only tricho- and only atrichoblast-bearing *wer myb23* and *cpc try* mutants, respectively. Cell-file-dependent sensitivity of PIN2 to BFA was visibly abolished in both classes of patterning mutants (Fig. 4G, H, I). While the relative fluorescence in BFA-bodies of control plants was 20% higher in trichoblast cells (Fig. 4J); PIN2 quantification in both mutants showed no detectable relative differences in BFA sensitivity of neighbouring epidermal cell files (Fig. 4K, L).

It is concluded that BFA-sensitive PIN2 trafficking and PIN2 abundance at the plasma membrane is not differentially controlled in epidermal patterning mutants. Our data suggest that tricho- and atrichoblast cells show cell-type-dependent differential regulation of turnover-based abundance of PIN2.

### Tricho- and atrichoblast mutants have altered sensitivity to exogenous auxin

**Fig. 5.**
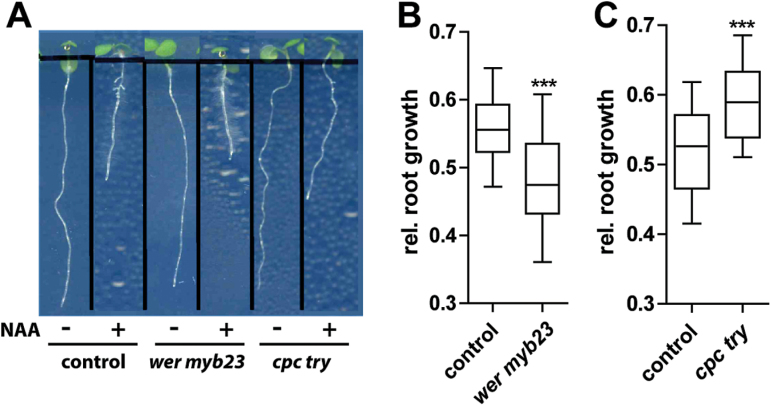
Differential auxin effect on root growth in epidermal patterning mutants. (A) Seedlings grown on 125nM auxin show root growth inhibition. (B) *wer myb23* plants show stronger auxin-mediated root growth inhibition than the control plants. (C) *cpc try* plants were partly resistant to auxin treatment, having significantly longer roots than the control plants. The data were statistically evaluated using Student’s *t* test. ***P <0,001; *n*=15.

It is very difficult to directly assess the contribution of tricho- and atrichoblast cells to root organ growth in wild-type seedlings. It was hypothesized that auxin responses might be altered in tricho- and atrichoblast mutants because they show lower and higher PIN2 abundance at the plasma membrane. In order to address the importance of these neighbouring epidermal cell files for auxin-dependent root growth, auxin-dependent root organ growth was investigated in the epidermal patterning mutants. Exogenous application of the synthetic auxin NAA at 125nM clearly affects root growth in wild-type seedlings (Fig. 5A). On the other hand *wer myb23* double mutants, having only trichoblast identity and lower epidermal PIN2 abundance, showed enhanced sensitivity to exogenous auxin applications (Fig. 5B). Conversely, *cpc try* double mutants, consisting only of atrichoblast cells and displaying higher PIN2 levels in epidermal cells, were partially resistant to auxin-dependent root-growth inhibition (Fig. 5C).

It is concluded that tricho- and atrichoblast patterning mutants show distinct sensitivity to exogenous auxin. This suggests that, in wild-type roots, both neighbouring epidermal cell files may contribute jointly to auxin-sensitive growth in wild-type seedlings.

### Trichoblast identity is required for defined gravitropic root growth

To investigate whether tricho- and atrichoblasts possibly contribute differentially to auxin-dependent development, gravitropic root growth was investigated and the epidermal patterning mutants were subjected to defined gravitropic stimuli.

**Fig. 6.**
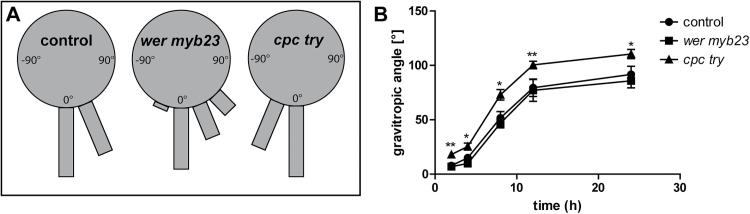
Epidermal patterning mutants show deviations in gravitropic responses. (A) After 16h gravistimulation (135°) roots were assigned to four sectors of 15° on a gravitropism diagram. The length of the bars in the diagram represents the percentage of seedlings assigned to the respective sector. *wer myb23* mutants show a slightly delayed and *cpc try* mutants an enhanced response to gravity. (B) Kinetics of the gravitropic response after 90° gravistimulation. The angle of every seedling was measured with respect to the initial (old) gravity vector after 2, 4, 8, 12, and 24h. The data were statistically evaluated using Student’s *t* test. *P <0.05, **P <0.01; *n*=12 for (A) and *n*=8–12 individual plants for (B).

*wer myb23* double mutants only possess trichoblast identity, including weaker epidermal PIN2 employment, and showed slightly slower gravitropic root growth when exposed to a 135° rotation (Fig. 6A). By contrast, following a 90° stimulation, the *wer myb23* double mutant was not distinguishable from wild-type seedlings (Fig. 6B).

*cpc try* double mutant roots only have atrichoblast identity (Löfke *et al.*, 2013), displaying higher PIN2 levels in meristematic epidermal cells and they showed hypergravitropic responses following 135° and 90° rotations (Fig. 6A, B). These data show that genetic interference with epidermal patterning genes has an impact on gravitropic root growth.

The cellular BFA responses in patterning mutants were altered compared to wild-type seedlings (Fig. 4G–L). Notably, root growth rates of *wer myb23* and *cpc try* mutants were similar to BFA-treated wild-type roots (see Supplementary Fig. S5A, B at *JXB* online). By contrast, it was noted that, already low BFA concentrations led to a strong agravitropic root growth, particularly in *cpc try* mutants (see Supplementary Fig. S5C at *JXB* online). These data pinpoint once again the joint, but possibly also distinct, requirements of the neighbouring epidermal cell files for gravitropic root growth.

Based on the gravistimulation assay and the BFA sensitivity data, it is hypothesized that trichoblast cell files may have higher importance for defined gravitropic root growth.

## Discussion

The root epidermis is a very interesting tissue as it establishes the rhizosphere interface and has the capacity to control root organ growth. In *Arabidopsis* the root epidermal cells are regularly spaced into shorter tricho- and longer atrichoblast cells. These neighbouring cell files may differentially contribute to root growth because the ectopic expression of the brassinosteroid receptor BRASSINOSTEROID-INSENSITIVE1 (BRI1) in trichoblasts promotes, but its overexpression in atrichoblasts inhibits root elongation (Fridman *et al.*, 2014). Auxin fluxes in the epidermis also control root organ growth (Luschnig *et al.*, 1998), but the underlying tissue requirements are largely unknown. Notably, the auxin influx carrier AUXIN RESISTANT1 (AUX1) has been shown to be preferentially expressed in atrichoblast cells (Swarup *et al.*, 2005). However, AUX1 activity in the lateral root cap and not its epidermal expression mainly contributes to gravitropic root growth (Swarup *et al.*, 2005). This study suggests the auxin transport rates could be distinct in neighbouring epidermal cell files. In the present study, PIN2 protein occurrence and trafficking was closely investigated in root epidermal cells. Transcriptional control of *PIN2* largely appeared non-differential in these neighbouring tissues (see Supplementary Fig. S2). By contrast, PIN2 proteins showed lower abundance in trichoblast cells. These data furthermore suggest that auxin biology is distinct in epidermal cell files. Vesicle trafficking inhibitors, such as BFA and wortmannin, interfere with cargo transition to the vacuole (Kleine-Vehn *et al.*, 2008) and induce stronger accumulation of PIN2 in trichoblast cells. In addition, higher GFP accumulation was observed in the vacuole in trichoblast cells of PIN2-GFP-, but not of ABCB19-GFP-expressing seedlings. In line with these findings, the genetic interference with the ubiquitin-based turnover of PIN2 completely abolishes its differential protein abundance at the plasma membrane in these cells. Our pharmacological, genetic, and live-cell imaging approaches accordingly suggest that PIN2 has a higher protein turnover in tricho- compared with atrichoblast cells. This distinct regulation of PIN2 trafficking towards the lytic vacuole for degradation is probably a consequence of cell-type specification, because epidermal-patterning mutants lost the distinct PIN2 trafficking and, subsequently, differential abundance at the plasma membrane. Our data show that auxin-related processes are partially distinct in tricho- and atrichoblasts. PIN2-dependent processes could provoke distinct contributions of tricho- and atrichoblast cells for root organ growth.

In this study, *cpc try* and *wer myb23* epidermal patterning mutants were used, because they lose the tricho- and atrichoblast identity already in the root meristem (Löfke *et al.*, 2013). It should be noted that these genes have so far been associated with epidermal patterning; however, epidermis unrelated functions are still possible. It is revealed here that the loss of tricho- or atrichoblast identity in *cpc try* or *wer myb23* mutants correlate with reduced and increased sensitivity to exogenous auxin, respectively. Hence, auxin sensitivity correlates with higher and lower abundance of PIN2 in epidermal cells. Even though it cannot be ruled out that epidermal patterning mutants affect multiple auxin-related and unrelated aspects, higher and lower PIN2 employment in epidermal cells could still be causal for root organ sensitivity to exogenous auxin applications. Exogenously applied auxin may not readily enter the stele in fully differentiated cells due to the endodermal diffusion barrier. Accordingly, most auxin, affecting root organ growth, may enter directly in the root tip. Notably, exogenous application of auxin in the nM range does not disrupt overall gravitropic root growth, suggesting that auxin transport can efficiently redistribute the exogenously applied auxin. Following this line of evidences, it is conceivable that PIN2-dependent transport capacity in the outermost root tissue may be protective for exogenous levels of its substrate. Accordingly, lower and higher abundance of PIN2 in epidermal patterning mutants could induce higher and lower sensitivity to exogenous auxins, respectively.

In conclusion, it is hypothesized that tricho- and atrichoblast jointly control auxin-dependent root organ growth in wild-type conditions. However, our previous work on epidermal cell size determination revealed that tricho- and atrichoblast cells show interdependent cell size regulation in wild-type seedlings (Löfke *et al.*, 2013). Therefore, it has to be concluded that not all root traits in these mutants depend on PIN2 function. In addition, it is possible that, not only cell size but also auxin sensitivity is interdependent in intact neighbouring wild-type epidermal cell files.

Remarkably, the loss of trichoblast identity in *cpc try* interrelates with hypergravitropic root growth. This finding is surprising, because *cpc try* mutants showed resistant root growth to exogenous auxin. Such a behaviour suggests that not auxin signalling but rather an auxin transport mechanism is responsible for these phenotypes. One possible explanation could be the higher overall abundance of PIN2 in *cpc try* roots compared with control roots (Fig. 4A, C). More PIN2 could lead to a more efficient transport of endogenous auxin and, hence, a faster response to gravity stimuli. Notably, *cpc try* roots not only respond faster, but also over-shoot, reflecting its additional defects in terminating the gravity response. PIN2 degradation could play a role in ceasing the gravitropic response (Baster *et al.*, 2013). Hence, it is tempting to speculate that reduced PIN2 turnover rates in *cpc try* could be linked to the observed overbending. However, it would require further investigation to fully validate such a conclusion. By contrast, the loss of atrichoblast identity in *wer myb23* seemingly had only mild effects on gravitropic root growth. Notably, gravitropic root growth of *cpc try* mutants was hypersensitive to the application of the vesicle trafficking inhibitor BFA. It is therefore tempting to speculate that the trichoblast cell files have a higher importance for gravitropic root growth.

It is concluded that epidermal cells show complex regulations of PIN2 auxin transport, jointly contributing to root organ growth and it is hypothesized that auxin-dependent processes, particularly in trichoblast cells, may be vital for defined gravitropic root growth. Further studies should also address other integrative growth regulatory aspects, such as the phytohormonal cross-talk of auxin and brassinosteroid in root epidermal cells. It is concluded that the root epidermis represents a suitable system to dissect such complex growth regulations and the integration of growth in neighbouring tissues.

## Supplementary data

Supplementary data can be found at *JXB* online.

Supplementary Fig. S1. ABCB19 displays homogeneous distribution at the PM of tricho- and atrichoblast cells.

Supplementary Fig. S2. Transcriptional PIN2 expression is not differentially regulated in tricho- and atrichoblast cells.

Supplementary Fig. S3. Distinct PIN2 trafficking in tricho- and atrichoblast cells.

Supplementary Fig. S4. Differential vacuolar targeting of PIN2-GFP in tricho- and atrichoblast cells.

Supplementary Fig. S5. Root length and BFA sensitivity of epidermal patterning mutants.

Supplementary Data
