# Supplementary material for: Tricho- and atrichoblast cell files show distinct PIN2 auxin efflux carrier exploitations and are jointly required for defined auxin-dependent root organ growth
Source: J Exp Bot. 2015 Jun 3;66(16):5103–12. doi: 10.1093/jxb/erv282 (PMC4513926; doi:10.1093/jxb/erv282)
Supplement: Supplementary Data [file supp_66_16_5103__index.html]

Tricho- and atrichoblast cell files show distinct PIN2 auxin efflux carrier exploitations and are jointly required for defined auxin-dependent root organ growth — Supplementary Data 

# Tricho- and atrichoblast cell files show distinct PIN2 auxin efflux carrier exploitations and are jointly required for defined auxin-dependent root organ growth

## Supplementary Data

Data files

- Supplementary Data - Supplementary Data
