# Supplementary material for: Tricho- and atrichoblast cell files show distinct PIN2 auxin efflux carrier exploitations and are jointly required for defined auxin-dependent root organ growth
Source: J Exp Bot. 2015 Jun 3;66(16):5103–12. doi: 10.1093/jxb/erv282 (PMC4513926; doi:10.1093/jxb/erv282)
Supplement: Supplementary Data [file supp_erv282_suppl_figures_L_fke___Scheuring.pdf]

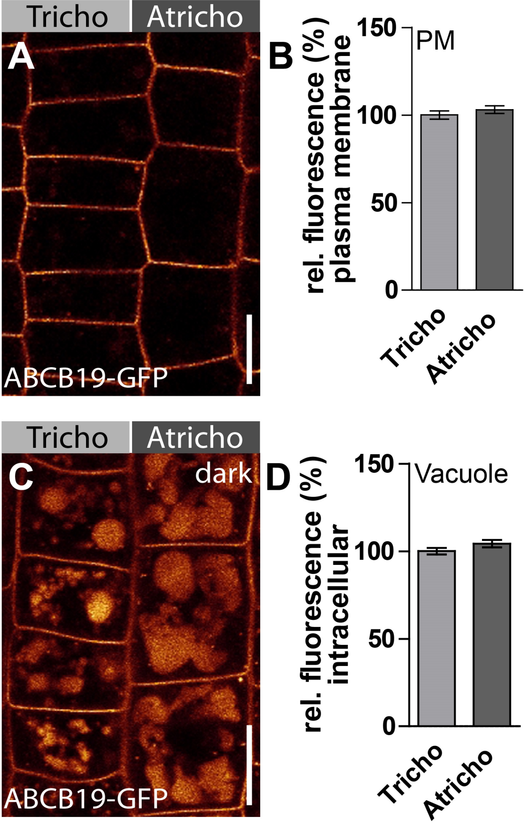

Figure S1. ABCB19 displays homogenous distribution at the PM of tricho- and atrichoblast cells. (A) ABCB19-GFP localization in tricho- and atrichoblast cells. (B) Quantification of plasma membrane signal intensity in tricho- and atricho-blast cells. (C) Accumulation of ABCB19-GFP in the vacuole (6-7 h dark treatment). (D) Quantification of intracellular signal (vacuole). The data was statistically evaluated using the student´s *t*-test. n =50 cells in 10 individual roots for B and n =25 cells in 5 individual roots for D. Scale bar: 10 µm.

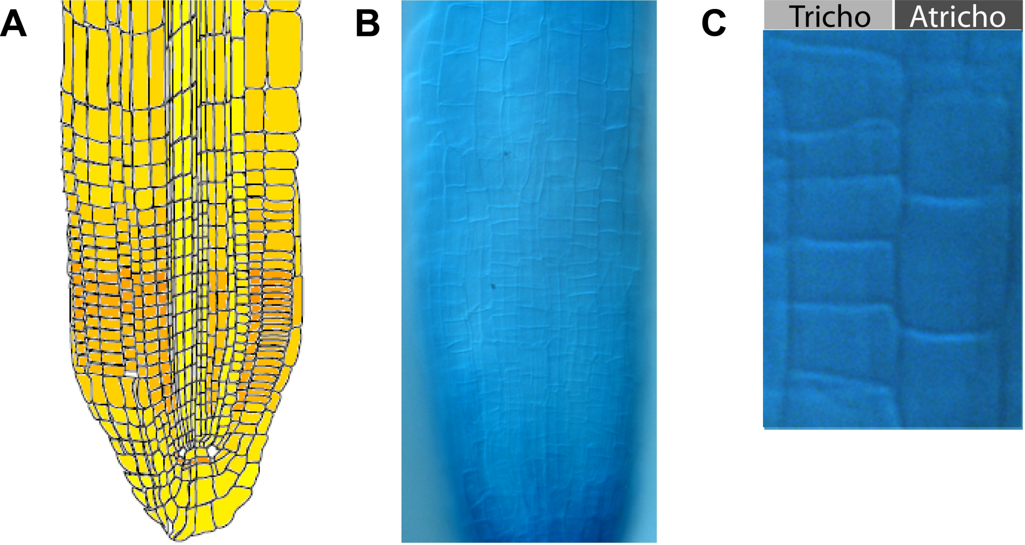

Figure S2. Transcriptional *PIN2* expression is not differentially regulated in tricho- and atrichoblast cells. (A) Micro array-based in situ analysis of *PIN2* expression in atrichoblast and trichoblast cell files (eFP browser: http://bar.utoronto.ca/efp/cgi-bin/efpWeb.cgi). (B) GUS-staining of plants expressing *pPIN2::GUS*. (C) Close-up of atrichoblast and trichoblast cells expressing *pPIN2::GUS*.

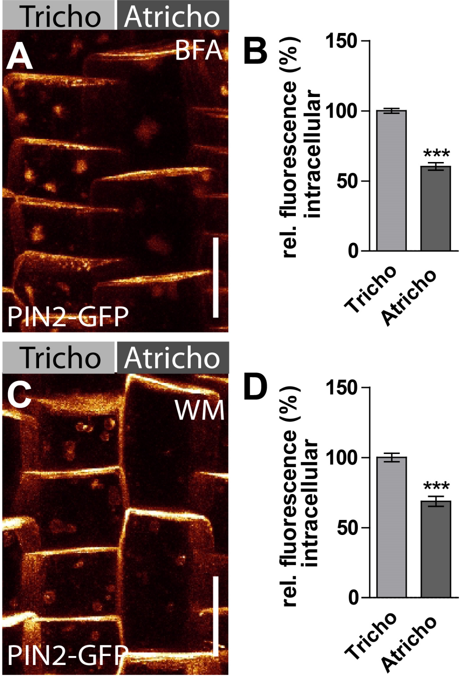

Figure S3. Distinct PIN2 trafficking in tricho- and atrichoblast cells. Maximum projection of PIN2-GFP labelled tricho- and atrichoblast cells treated with (A) BFA (50 µM; 90 min) or (C) WM (30 µM; 2 h). (B) Mean grey value quantification of entire cell area (intracellular) in BFA treated seedling of tricho- and atrichoblast cells. (D) Mean grey value quantification of entire cell area (intracellular) in WM treated seedling of tricho- and atrichoblast cells. The data was statistically evaluated using the student´s *t*-test. ***$p < 0.001$; n =25 cells in 5 individual roots. Scale bar: 10 µm.

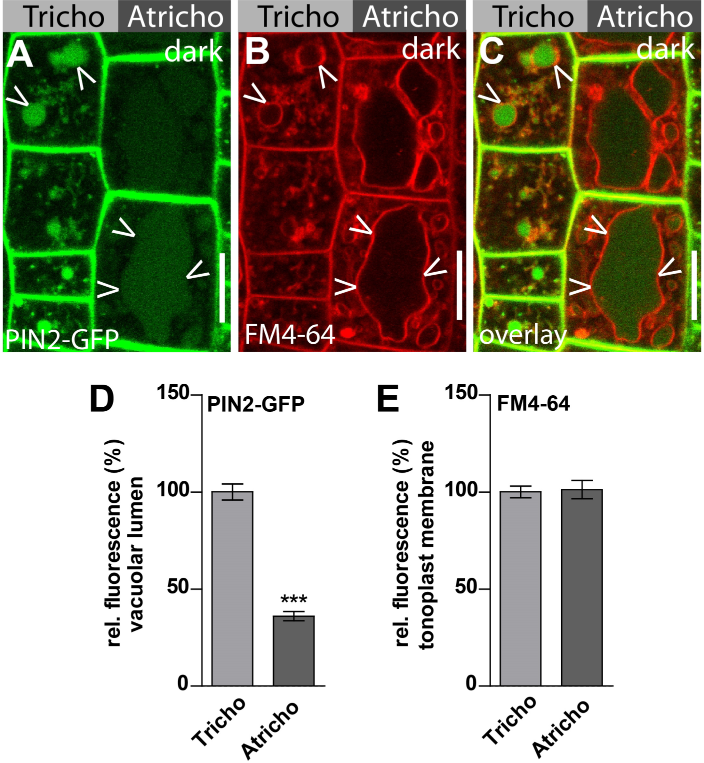

Figure S4. Differential vacuolar targeting of PIN2-GFP in tricho- and atrichoblast cells. (A-E) Comparison of PIN2-GFP and FM4-64 accumulation in the vacuolar lumen or tonoplast membrane, respectively. (A-B) PIN2-GFP (A) accumulation in the vacuole (dark, 5 h) and simultaneous FM4-64 staining (B) of tonoplast membrane. (C) Merged picture of A and B depicts GFP signals surrounded by the FM4-64 stained tonoplast membrane. (D) Quantification of mean grey values of GFP signal intensity in the vacuolar lumen of tricho- and atrichoblast cells. (E) Quantification of mean grey values of FM4-64 signal intensity at the tonoplast of tricho- and atrichoblast cells. Arrow head illustrate the position of the vacuole. The data was statistically evaluated using the student´s *t*-test. ***$p < 0.001$; n =25 cells in 5 individual roots. Scale bar: 10 µm.

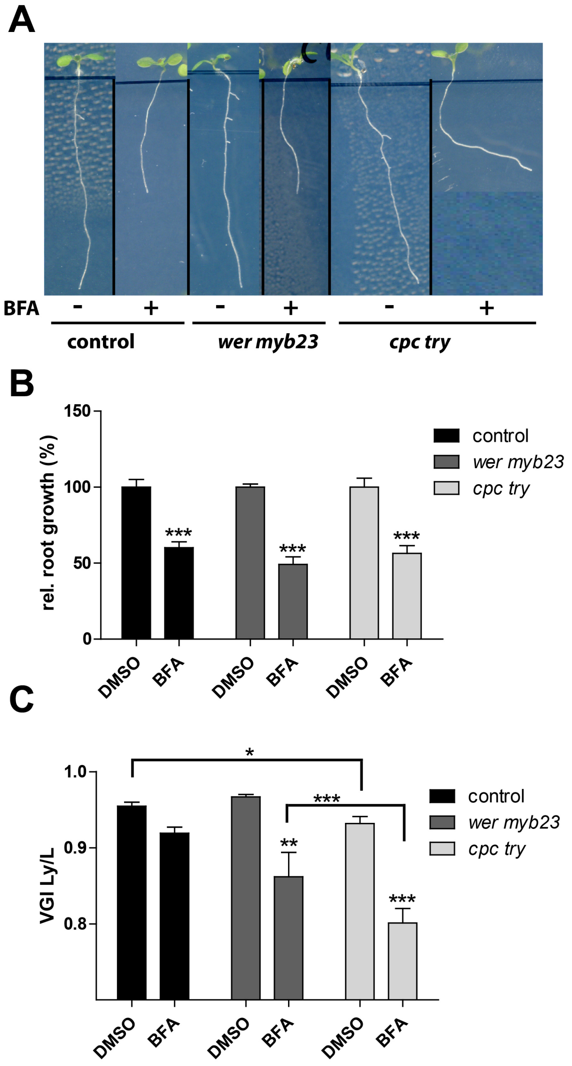

Fig. S5. Root length and BFA sensitivity of epidermal patterning mutants. (A) Seedlings were germinated vertically on ½ MS plates supplemented with 5 µM BFA or DMSO as a solvent control. After 6-7 days the root length was determined (B) Quantification of root length displays a strong reduction in length if grown on 5 µM BFA. (C) In order to take the growth direction into account the vertical growth index (VGI) was calculated for plants grown on BFA. *cpc try* plants show a significantly lower VGI than control plants and *wer myb23*. The data was statistically evaluated using the student´s *t*-test. *p <0.05, **p <0.01, ***p < 0.001; n =10.
